# Epicardial Fat and Its Association with Cardiovascular Risk: A Cross-Sectional Observational Study

**DOI:** 10.4103/1995-705X.76801

**Published:** 2010

**Authors:** Farouk Mookadam, Ramil Goel, Mohsen S. Alharthi, Panupong Jiamsripong, Stephen Cha

**Affiliations:** Division of Cardiovascular Diseases, Mayo Clinic Scottsdale, Minnesota, USA; 1 Division of Biomedical Statistics and informatics, Mayo Clinic Rochester, Minnesota, USA

**Keywords:** Cardiovascular risk, epicardial adipose tissue, myocardial dysfunction, metabolic syndrome, visceral adipose tissue

## Abstract

**Background:**

The association between visceral obesity and cardiovascular risk has been well described. Some studies show a proportional relationship between the presence of visceral obesity and epicardial fat. Measuring the amount of epicardial adipose tissue (EAT) can be a novel parameter that is inexpensive and easy to obtain and may be helpful in cardiovascular risk stratification. However, the relationship between epicardial fat and cardiac function and that between epicardial fat and cardiac risk factors is less well described.

**Objectives:**

To evaluate the association between echocardiographic epicardial fat and the morphologic and physiologic changes observed at echocardiography and to evaluate the association between epicardial fat and cardiac risk factors. A cross-sectional study of 97 echocardiographic studies (females, n = 42) was conducted. Two groups were identified: epicardial fat ≥ 5 mm (group I) and <5 mm (group II).

**Results:**

Epicardial fat >5 mm was associated with LA enlargement, with lower ejection fraction, increased left ventricular mass, and abnormal diastolic function. On a multivariable regression analysis, all these parameters also correlated individually with EAT thickness independent of age. Hyperglycemia (DM), systolic hypertension, and lipid parameters for metabolic syndrome showed a trend for positive association, but this was not statistically significant. The association was not significant even for higher cutoff limits of EAT thickness.

**Conclusion:**

Epicardial fat >5 mm is associated with cardiac abnormalities on echocardiography. This is a sensitive assessment of body fat distribution, is easily available at echocardiography, and is simple to acquire at no added cost. Further studies looking at the appropriate cut-off thickness of EAT and the sites of measurement to be used are needed. Comparison of this simple and inexpensive measure with other measures of obesity, such as waist-hip ratio, body mass index, Dexa scan of visceral fat, and magnetic resonance imaging of visceral, are needed.

## INTRODUCTION

Epicardial adipose tissue (EAT) is part of the visceral adipose tissue (VAT) distributed around the viscus or hollow muscular organs of the body. EAT owes its embryologic origin to the splanchnopleuric mesoderm which also gives rise to mesenteric and omental fat.[1] VAT is now well established as being associated with the development of metabolic syndrome and coronary artery disease.[2] The mechanism of these effects of VAT are not entirely understood, but could be mediated by release of free fatty acids causing direct ‘lipotoxicity.’[3,4] Adipose tissue, especially the VAT, also acts as an endocrine organ, releasing numerous proinflammatory and proatherogenic cytokines and hormones affecting endothelial function.[5,6]

Increased body mass index (BMI) and waist-hip ratio are also useful markers of cardiovascular risk, but are not specific as they are confounded by subcutaneous adipose tissue and lean body mass.[7] Many imaging modalities, including computed tomography (CT) and multi slice magnetic resonance imaging (MRI), have been used to image and quantify deposits of VAT and also have been shown to correlate well with development of metabolic syndrome.[8–10] There is an increasing rationale for estimating VAT with novel imaging techniques such as MRI, MR spectroscopy, and ultrasound for cardiovascular risk stratification.[11] However, these imaging techniques are expensive and not routinely available and may have contraindications in select patient populations.

Transthoracic echocardiography has been validated as an easy and reliable method to quantify the presence of VAT by measuring the EAT, which correlates very well with the presence of general VAT.[12–14] The relationship of EAT to cardiac function and cardiovascular risk factors is not well understood and needs more study before it can be used as a tool for routine clinical assessment.

In this study, we sought to examine the relationship between the thickness of EAT with cardiac morphologic and physiologic changes as seen on echocardiography. We also investigated the association of EAT with conventional cardiovascular risk factors.

## MATERIALS AND METHODS

### Subjects

Consecutive patients presenting to the noninvasive hemodynamic laboratory at our institution were eligible for the study if informed consent was provided. A total of 97 patients (females, n = 42) were prospectively recruited in the study after informed consent and Institutional Review Board approval. Patients were divided into the following two groups according to the epicardial thickness: epicardial fat <5 mm (group I) and ≥5 mm (group II). The reason for arriving at the figure of 5 mm was that the mean thickness of EAT for this cohort was 5.3 mm and the median was 4.9 mm.

### Echocardiographic study

**Figure 1:**
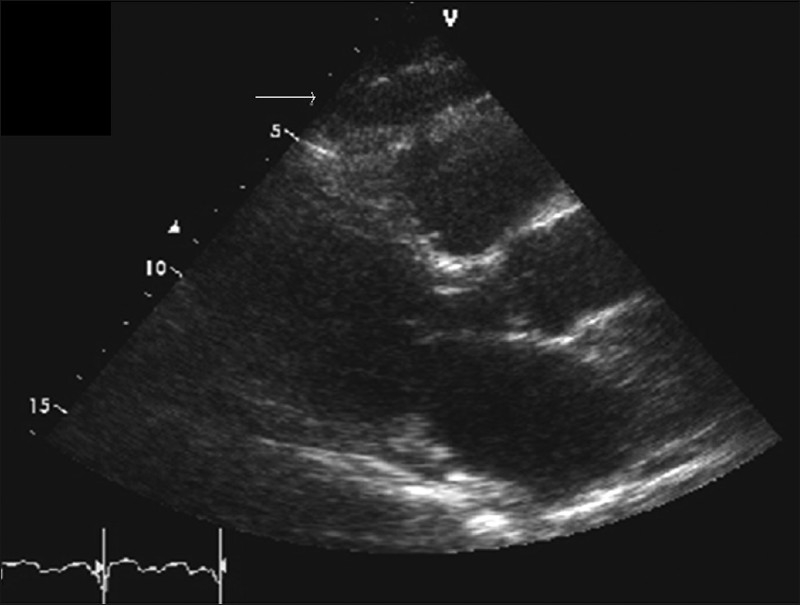
Long-axis parasternal view from transthoracic echocardiogram. The arrow points to the epicardial fat over the free wall of the right ventricle, appearing as the echo-free space between the outer layer of the myocardial wall and the visceral layer of the pericardium

Each subject underwent detailed transthoracic two-dimensional, M-mode Doppler, and tissue Doppler imaging echocardiography was performed with a GE instrument (GE Healthcare, Milwaukee, WI) by standard techniques as described earlier,[15,16] with subjects in the left lateral decubitus position. Echocardiograms were uploaded digitally to an online system (ProSolv CardioVascular, Indianapolis, IN) for measurement and analysis. Echocardiograms were interpreted by experienced echocardiologists. Readers were blinded to the subjects and to results of each other. Epicardial fat thickness was measured on the free wall of the right ventricle from both parasternal long- and short-axis views at mid ventricle during end diastole (marked by the R wave on the ECG recording). The maximum values at each site were measured, and the average value was considered. EAT is defined as the echo-free space between the outer layer of the myocardial wall and the visceral layer of the pericardium and measured as such [Figure 1].[14,17]

Other echocardiographic measures were measured per American Society of Echocardiography guidelines including left atrial volume (LAV), using area length method indexed to body surface area; ejection fraction (EF) was measured using volumetric biplane Simpson method. Serum fasting glucose and fasting lipid profile were obtained through a certified laboratory, BMI was calculated from patient morphometrics, blood pressure (BP) was recorded by cuff measurement from the brachial artery at the time of study, and left ventricular mass index (LVMI) and diastolic parameters (E, A, deceleration time [DT], E/A ratio, IVRT, and E/e’) were compared in the two groups [Table 1].

**Table 1:** Clinical and echocardiographic characteristics of both groups

Variable	Group I Epi. F<5 mm (N = 52)	Group II Epi. F>5 mm (N = 45)	*P* value
Age (yrs)	60.29 ± 18.32	73.56 ± 6.44	<0.001
Height(cm)	172.9 ± 11.14	170.4 ± 10.03	0.25
Weight(kg)	85.03 ± 22.27	83.33 ± 17.89	0.68
BMI (kg/m2)	28.38 ± 6.68	28.49 ± 4.53	0.93
SBP (mmHg)	128 ± 19.74	132.1 ± 16.97	0.28
DBP (mmHg)	72.83 ± 10.53	72.29 ± 9.51	0.79
HR (beats/min)	69.71 ± 13.84	71.07 ± 14.84	0.64
EF (%)	61.73 ± 8.79	56.91 ± 11.75	0.024
LV mass (g)	185.9 ± 71.61	218.4 ± 92.34	0.07
LVMI (g/m2)	92.33 ± 27.17	110.2 ± 36.43	0.01
LAVI (AL)	35.15 ± 13.79	39.63 ± 17.54	0.16
CO (l/min)	5.14 ± 1.28	5.11 ± 1.38	0.89
CI (l/min/m2)	2.63 ± 0.63	2.63 ± 0.78	1
E Vel (m/s)	0.91 ± 0.28	0.88 ± 0.43	0.69
A Vel (m/s)	0.72 ± 0.19	0.91 ± 0.25	<0.001
E/A	1.27 ± 0.44	0.89 ± 0.36	<0.001
Diastology (E/e’)	14.39 ±10.45	16.92 ±9.18	0.2205
DT (ms)	202.7 ± 45.08	223.7 ± 70.98	0.09
FBS (mg/dl)	107.7 ± 26.68	112 ± 20.73	0.39
T. cholestrol (mg/dl)	176.9 ± 37.07	171.4 ± 31.21	0.47
LDL (mg/dl)	95.41 ± 31.02	89.34 ± 26.71	0.35
HDL (mg/dl)	60.1 ± 20.29	56.24 ± 14.27	0.32
TG (mg/dl)	108.6 ± 52.01	129.8 ± 56.68	0.08

A Vel = Late filling wave velocity across mitral valve due to atrial contraction; BMI = body mass index; CO = cardiac output; CI = cardiac index; DBP = diastolic blood pressure; DT = deceleration time; Epi.F = epicardial fat; E Vel = peak mitral flow velocity of early rapid filling wave; E/e’ = ratio of early diastolic wave across mitral valve from pulse wave Doppler across mitral wave divided over the e’ wave which corresponding to the early diastolic wave from tissue Doppler imaging at the medial mitral annulus; EF = ejection fraction; FBS = fasting blood sugar; HR = heart rate; HDL = high density lipoprotein; LAVI = left atrial volume index; LDL = low density lipoprotein; LVMI = left ventricle mass index; SBP = systolic blood pressure; TG = triglyceride; T. cholesterol = total cholesterol.

Some investigators have measured EAT in end systole and the issue of the timing of EAT measurement is not settled. However, the EAT measurements by CT and MRI are made in end diastole and there is precedence of measuring EAT in end diastole by echocardiography too. Perhaps, further studies are needed, looking at the best time point of measurement which correlates with the EAT volume by CT or MRI.

### Statistical analysis

Data were summarized as mean ± standard deviation. The two groups (group I EF <5 mm and group II EF ≥5 mm) were compared for each variable in Table 1 by two-sample t-test. Univariate and multivariate regression analysis were performed to look for correlation of EAT thickness with age, LVMI, EF, and E/A ratio. Any P value of less than 0.05 was considered statistically significant. As shown in Table 2, each variable was checked to see if it correlated with EAT univariately, and after age adjustment. Age, male gender, EF, LV mass, E/A (mitral inflow Doppler), and DT were selected in the multivariate model. Only age, LVMI, and E/A remained after backward elimination process as shown in Table 3. Interclass correlation coefficient (ICC) was calculated with a 95% confidence interval to evaluate the reliability of the EAT measurement. ICC had adopted the same interpretation as the kappa statistics: ICC >0.75 was considered as excellent, 0.4 to 0.75 as good, and ICC <0.4 as poor[18] All statistical analyses were performed by SAS 9.1.3 software (SAS Institute Inc, Cary NC).

**Table 2:** Univariate and age-adjusted regression model analyses

Variable	Univariate analysis			Univariate analysis with age adjustment		
	Parameter estimate	Standard error	*P* value	Parameter estimate	Standard error	*P* value
BMI	0.0567	0.0491	0.2507	0.0780	0.0413	0.0619
SBP	0.0229	0.0145	0.1174	0.0002	0.0131	0.9854
DBP	0.0120	0.0274	0.6633	0.0194	0.0232	0.4041
HR	0.0109	0.0192	0.5727	0.0065	0.0164	0.6907
EF	-0.0593	0.0256	0.0225	-0.0275	0.0229	0.2333
LV mass	0.0090	0.0035	0.0117	0.0070	0.0031	0.0258
LVMI	0.0255	0.0088	0.0046	0.0163	0.0078	0.0387
CO	0.1153	0.2171	0.5967	0.3549	0.1807	0.0527
CI	0.0796	0.4171	0.8492	0.3114	0.3511	0.3777
E Vel	-0.7754	0.7698	0.3164	-0.5024	0.6503	0.4419
A Vel	4.9407	1.1609	<0.0001	2.0152	1.1632	0.0874
E/A	-3.0540	0.5982	<0.0001	-1.1177	0.6925	0.1109
DT	0.0129	0.0046	0.0057	0.0061	0.0041	0.1469
LAVI	0.0280	0.0171	0.1052	-0.0020	0.0156	0.8966
FBS	0.0152	0.0113	0.1824	0.0025	0.0101	0.8063
T. Cholestrol	-0.0086	0.0087	0.3281	0.0022	0.0077	0.7734
LDL	-0.0137	0.0103	0.1867	-0.0008	0.0092	0.9274
HDL	-0.0137	0.0171	0.4253	-0.0063	0.0146	0.6679
TG	0.0096	0.0054	0.0772	0.0088	0.0045	0.0561

A Vel = late filling wave velocity across mitral valve due to atrial contraction; BMI = body mass index; CO = cardiac output; CI = cardiac index; DBP = diastolic blood pressure; DT = deceleration time; E Vel = peak mitral flow velocity of early rapid filling wave; EF = ejection fraction; FBS = fasting blood sugar; HR = heart rate; HDL = high density lipoprotein; LAVI = left atrial volume index; LDL = low-density lipoprotein; LVMI = left ventricle mass index; SBP = systolic blood pressure; TG = triglyceride; T. cholesterol = total cholesterol

**Table 3:** Multivariate regression model

Variable	Parameter estimate	Standard error	*P* value
Proposed model (N = 65)			
Age	0.06118	0.02292	0.0098
Male gender	0.3723	0.56221	0.5105
EF	-0.03602	0.02916	0.2217
LVMI	0.01511	0.00949	0.1166
E/A ratio	-1.34914	0.83902	0.1133
DT	0.0064	0.00508	0.2125
Final model after backward elimination			
Age	0.0707	0.02242	0.0025
LVMI	0.01901	0.00883	0.0353
E/A ratio	-1.50531	0.77274	0.056

EF = ejection fraction; LVMI = left ventricle mass index; DT = deceleration time

## RESULTS

The mean age of patients was 66.44 ± 5.54 years. The proportion of males was 60% (57). EAT thickness > 5 mm was also significantly associated with older patients’ age (73.5 *vs* 60, *P* = <0.01). The EAT thickness was not affected by gender. There was no significant difference between males and females in all measured characteristics studied, except heart rate and total cholesterol. EAT thickness > 5 mm was associated with LA enlargement with LAVI (40 *vs* 35.5 cc/m^2^, *P* = 0.16), with lower EF (56.7 *vs* 61.8%, *P* = 0.024), increased LVMI (110 vs 92 g/m^2^, *P* = 0.01), and abnormal diastolic function demonstrated by a higher E/e’ ratio (16.9 *vs* 14.4, *P* = 0.2205). Although higher epicardial fat thickness was associated with older age, on a multivariable regression analysis, the relationship of EAT thickness and LVMI, EF, and E/A ratio was shown to be independent of age.

EAT thickness > 5 mm was also associated with higher fasting glucose levels (serum glucose, 112 *vs* 107.7 mg/dl, *P* = 0.39) and systolic hypertension (132 *vs* 128 mmHg, *P* = 0.28), and the lipid parameters for metabolic syndrome also showed a trend for positive association. However, the association of EAT thickness ≥5 mm with hyperglycemia, hypertension, and dyslipidemia was statistically nonsignificant [Table 1]. This association did not show statistical significance even when higher cut-off values of EAT thickness at 6 and 7 mm were used. Notably, these results were seen despite a similar BMI (28.49 *vs* 28.38) and BSA (1.95 *vs* 1.98 m^2^) in both groups.

## DISCUSSION

There is an association between obesity and adaptive modifications in cardiac morphology and function.[19–21] The presence of visceral obesity, even in clinically nonobese patients seems to confer an increased risk of higher LV mass and diastolic dysfunction.[22,23] EAT seems to be a marker of the overall content of VAT in the body and in addition has anatomical proximity to the heart. It is thus reasonable to expect EAT to be closely associated with derangements in cardiac morphology and function. Autopsy studies have shown a parallel increase in LV mass with increasing EAT, which was noted to be independent of ischemia.[24] Echocardiography data also show a correlation between EAT mass and LV mass.[25] In light of available data, our study further bolsters the association of increased EAT with increased LV mass. The mechanism for such an association is still unclear, but a causal link could be present from a combination of different factors. The following four proposed mechanisms acting in varying degrees are possible:

1. The epicardial fat pad constitutes a mechanical load on the heart, which has to be moved with every cardiac cycle and could lead to compensatory remodeling.[26]
2. The release of adipokines from the EAT can locally induce cardiac remodeling.[26] Human EAT is metabolically active and secretes various cytokines including TNF-alpha, IL-1, IL-6, and monocyte chemotactic protein-1.[27] It is conceivable that these mediators act in a paracrine fashion to directly induce deleterious changes in myocardial morphology. Increasing evidence seems to point toward EAT as a metabolically active tissue modulating the adjacent myocardial tissue.[28] Therefore, rather than being a benign bystander, EAT seems to affect the heart in a significant way.
3. Another interesting mechanism may be the direct ‘lipotoxic’ effects of intracellular accumulation of triglycerides and byproducts of lipid metabolism like ceramides.[4] It is possible that free fatty acids could diffuse directly from the EAT into myocardial cells exacerbating myocardial steatosis and lipotoxicity, leading to adverse structural and functional cardiac adaptations.[28] If an extreme form of this condition can cause cardiomyopathy, it is conceivable that milder versions of this condition exist, characterized by subclinical alterations in the form and function of the heart.
4. EAT also can affect the heart through its systemic effects. It is closely associated with insulin resistance and glucose intolerance, and these conditions could also predispose independently to derangements in LV mass and function.[29,30]

We also demonstrate that increased EAT is related to diastolic dysfunction and left atrial enlargement. This has been shown in a previous study by Iacobellis *et al*.[31] The etiology of the LV diastolic dysfunction may be related to above previously mentioned factors and the atrial enlargement may be secondary to the increased filling pressures from diastolic dysfunction and the epicardial fat pad itself impairing LV filling.[32]

The presence of increased VAT in the abdomen is strongly associated with the metabolic syndrome.[33,34] EAT is also emerging as an important marker for metabolic syndrome. The presence of EAT as measured by echocardiography also showed good correlation with waist circumference, fasting insulin, and diastolic BP.[14] In a recent study, the median values of end systolic measurement of EAT >9.5 mm for men and >7.5 mm for women was associated with the metabolic syndrome.[35] A recent study showed that EAT thickness of greater than 12.4 mm correlated with the presence of at least two markers of metabolic syndrome including hypertension, dyslipidemia, and hyperglycemia.[36] This study is in press at the time of writing and used multi-detector CT (MDCT) to measure EAT thickness at the left atrioventricular (a-v) groove. Our results seem to parallel these findings. The results for metabolic syndrome are statistically nonsignificant due to the lower threshold of EAT thickness determined by us. In the MDCT study,[36] the fat pad in the a-v groove is measured, and this is the thickest fat pad around the heart; no clear reasons were provided. In the prior studies of Iacobellis *et al*.,[35] EAT is measured during systole and may exaggerate the EAT if the adipose tissue is cut tangentially; furthermore, this is the reason for higher EAT cut-off points as compared with those used in our study where diastolic frames are used for measurement.

The traditional Framingham risk factors have fairly good predictive accuracy for cardiovascular disease but newly emerging risk factors such as C-reactive protein, lipoprotein (a), and carotid artery intimal thickness might have increased clinical utility in future.[37] The additional use of imaging tools might be synergistic to the available and emerging biomarkers.[38]

There are various imaging modalities like MRI and CT which are currently gold standard for measuring VAT content.[26] These however are expensive, and are not routinely performed in a typical cardiac patient. However, echocardiogram provides a relatively inexpensive means to measure and quantify an important component of VAT, the EAT, which may have similar influence on cardiovascular risk profile. The additional measurement of EAT pad thickness takes no extra-specific training and adds minimally to the time of a regular echocardiography procedure. This modality however needs further testing and validation before it can be widely used and placed in clinical algorithms of risk stratification.

## CONCLUSION

Obesity is a growing epidemic worldwide and is associated with cardiovascular risk, with the development of the metabolic syndrome, diabetes mellitus II, and sleep-disordered breathing. Visceral adiposity is a marker of increased risk but not readily assessed and may be costly to measure. Epicardial fat thickness ≥5 mm is associated with cardiac morphologic abnormalities on echocardiography. This may be a more sensitive assessment of body fat distribution. It is easily available at echocardiography and simple to acquire at no added cost and training. Further studies on the presence of epicardial fat ≥5 mm and cardiovascular outcome are needed. Standardization of measurement and sensitivity of EAT in predicting CV risk are needed. Comparison of this simple and inexpensive measure with waist-hip ratio, BMI, Dexa scan of visceral fat, and MRI of visceral fat are needed before this modality of risk stratification becomes routine.
